# Terminology describing medical imaging professionals

**DOI:** 10.1002/jmrs.364

**Published:** 2019-10-27

**Authors:** Warren Reed

**Affiliations:** ^1^ Medical Image Optimisation and Perception Group Discipline of Medical Imaging Science The University of Sydney Sydney NSW Australia

Thank you to Professor Davidson for the complimentary remarks concerning our article^1^. It is very encouraging that this type of article is so appreciated.

As to terminology, the initial selection of the term ‘technician’, as used in the article, was simply an attempt to be more inclusive as it was considered that the title’s ‘radiographer’ and/or ‘radiologist technologist’ may not always be directly applicable to adequately describe the profession throughout all the countries represented by the International Society of Radiographers and Radiological Technologists (ISRRT) across the world^2^. Interestingly, there appear numerous different titles for medical imaging professionals and radiographers across Europe alone often consistent with the diversity in each country in language and culture including their academic qualifications and job description^2^. Also, several non‐English‐speaking countries use the title 'radiology technicians' whereas in some other English‐speaking countries they use the word 'tecs' which can describe both technologists and technicians^2^. However, the titles ‘radiographer’ and ‘radiologic technologist’ are recognised as the correct terminology as used in Australia, UK, Ireland and the USA as well as many other countries.

On reflection, we are in total agreement with the points raised and the word ‘technicians’ has been replaced with ‘medical imaging professionals’ in the first instance and ‘technicians’ is replaced by ‘radiographers’ in the second as we fully appreciate it is very important to be precise about our professional role, educational background and scope of practice. These clarifications have further strengthened our article and we would like to thank Professor Davidson for bringing them to our attention.

## References

[1] Hooper T , Eccles G , Milliken T , Mathieu‐Burry JR , Reed W . Dose reduction in CT imaging for facial bone trauma in adults: A narrative literature review. J Med Radiat Sci 2019; 66: 122–32. 30706691 10.1002/jmrs.319 PMC6545476

[2] "Tecs" Vs Radiographers [Internet]. ISRRT. 2018 [cited 19 Sept 2019]. Available from: https://www.isrrt.org/blog/tecs-vs-radiographers?fbclxml:id=IwAR1PZb4AE5ryqE8jVPJZfVv6XrAYdBbKwfDRIl2aMqKS6NbzfEQjoU10giw.

